# Anorexia Nervosa and Celiac Disease in an Adult: A Case Report

**DOI:** 10.7759/cureus.30494

**Published:** 2022-10-19

**Authors:** Antonios Tsakiris, Styliani Papantoniou, Panagiotis Kouvatsos, Charalampos Tamvakos, Stavros Antonopoulos

**Affiliations:** 1 Department of Internal Medicine and Diabetes Center, Tzaneio General Hospital of Piraeus, Piraeus, GRC; 2 Department of Internal Medicine, Arvika Hospital, Arvika, SWE; 3 Department of Cardiology, Athens General Hospital 'Elpis', Athens, GRC

**Keywords:** chronic diarrhea, alt:ast ratio, autoimmunity, celiac disease, anorexia nervosa

## Abstract

Previous studies suggest an association between celiac disease and anorexia nervosa. Research has mainly focused on children and adolescents, and studies among adults are limited. The similar clinical manifestations that characterize both diseases can complicate the diagnosis, and a thorough diagnostic workup is necessary. A focused medical history remains the cornerstone of diagnosis. A delayed diagnosis can lead to a worse quality of life and severe complications. We present the case of a 43-year-old woman with anorexia nervosa who was thereafter diagnosed with celiac disease. The later diagnosis occurred after a long period of persistent diarrhea. Based on the patient’s history of autoimmune disease, celiac disease was suspected. Our case highlights the importance of additional work-up in patients with anorexia nervosa who have persistent gastrointestinal symptoms. A further investigation should be based on the medical history, clinical presentation, and laboratory findings.

## Introduction

The word anorexia originates from the Greek words an- (ἀν-, prefix denoting negation) and orexis (ὄρεξις, "appetite"), and means pathological loss of appetite [1]. According to DSM-5 (a resource used in the diagnosis of mental disorders), the diagnosis requires each one of the following: restriction of energy intake relative to requirements leading to low body weight, intense fear of gaining weight, or persistent behaviors that interfere with gaining weight, and disturbance in the way a person's weight or body shape is experienced or a lack of recognition about the risks of the low body weight [2]. Anorexia nervosa is a heterogeneous disease and requires appropriate individualized treatment for each patient [3]. An individualized treatment plan involves restoration of weight and nutritional status and psychotherapy [4].

Celiac disease is a long-term immune-mediated inflammatory disorder, primarily affecting the small intestine, where genetically predisposed individuals develop intolerance to gluten, present in foods such as wheat, rye, and barley [5]. Diagnosis is based on clinical presentation, serologic tests, intestinal biopsies, and sometimes targeted genetic testing. The first step of the diagnostic approach is the immunoglobulin (Ig)A-anti-tTG (tissue transglutaminase) test and the measurement of total IgA levels. A small bowel biopsy is necessary for patients with positive serology to confirm the diagnosis [6]. A strict gluten-free diet is the only effective treatment for celiac disease [5,6]. 

Various studies suggest that anorexia nervosa is bi-directionally related to autoimmune diseases [7]. Moreover, patients with celiac disease are at increased risk of developing eating disorders [8]. Although the pathophysiologic mechanism is not fully understood and several hypotheses regarding genetic and immunological risk factors have been proposed [9].

Herein, we describe the case of a 43-year-old woman diagnosed with anorexia nervosa and with a history of chronic diarrhea. A further diagnostic investigation based on the patient’s medical history led to a diagnosis of celiac disease. A small bowel biopsy confirmed the diagnosis.

## Case presentation

A 43-year-old woman was admitted to the emergency department of a health center with a four-month history of chronic diarrhea, 10-15 episodes/day, and fatigue. She received loperamide and probiotics, but the symptoms persisted. Her past medical history was remarkable for a history of hyperthyroidism, for which she underwent treatment with radioiodine and was diagnosed with anorexia nervosa five years ago. She was under the treatment of SSRIS and cognitive-behavioral therapy, but frequent relapses had occurred. Physical examination revealed an extremely underweight woman with a body mass index of 14.2, who was affected and hypovolemic with dry mucous membranes, poor skin turgor, and prolonged capillary filling. She was alert and oriented, but with a deficit in attention and concentration. Her initial vitals were a rectal temperature of 36.5^o^C, a heart rate of 104 beats per minute, and a blood pressure of 90/60 mm Hg. Laboratory analysis revealed hypochromic microcytic anemia with low ferritin levels, mild hyponatremia, mild hypokalaemia, elevated transaminases, and prolonged international normalized ratio (INR) (Table 1).

**Table 1:** Laboratory findings on admission MCV: mean corpuscular volume, MCH: mean corpuscular hemoglobin, INR: international normalized ratio

Variable	Results	Normal range
Serum Alanine transaminase (ALT)	103 U/mL	5-40 U/mL
Serum Aspartate transaminase (AST)	91 U/mL	5-35 U/mL
Urea	58 mg/dL	14-50 mg/dL
Creatinine	1.1 mg/dL	0.6-1.1 mg/dL
Hemoglobin	10.2 g/dL	12-16 g/dL
MCV	71 fL	80-100 fL
MCH	23 pg	27-34 pg
Ferritin	18 μg/L	20-270 μg/L
Sodium	131 mmol/L	135-150 mmol/L
Potassium	3.3 mmol/L	3.5-5 mmol/L
INR	1.4	0.8-1,.2

An ultrasound examination of the liver and gallbladder did not reveal any pathological findings, while the laboratory assessment of thyroid function showed subclinical hypothyroidism. She received normal saline and multivitamin preparation and her clinical condition was stabilized. She started gradually on a high-calorie diet and recovered, but without a decrease in the frequency of diarrhea. The persistence of diarrhea raised suspicion that the cause of the patient's symptoms was not anorexia nervosa. We performed further workup to rule out other diseases that could coexist. Screening for celiac disease was performed, considering the autoimmune background (Table 2).

**Table 2:** Serologic tests for Celiac disease Ig: immunoglobin

Serologic test	Results	Normal range
Anti-deamidated gliadin peptide IgA (Anti-GDP/AGA IgA)	203.2 U/mL	< 10 U/mL
Anti-deamidated gliadin peptide IgG (Anti-GDP/AGA IgG)	199.2 U/mL	< 10 U/mL
Anti-tissue transglutaminase Antibodies IgA (h-tTG IgA)	185.2 U/mL	< 10 U/mL
Anti-tissue transglutaminase Antibodies IgG (h-tTG IgG)	2.6 U/mL	< 10 U/mL
Serum IgA	424 mg/dL	70-400 mg/dL

A duodenal biopsy was necessary to confirm the new diagnosis. The biopsy revealed enteropathy with villous atrophy (type Marsh 3) and confirmed that the patient was suffering from celiac disease. She started on a gluten-free diet and after three months; the patient gained weight, her diarrhea subsided, her liver tests were normal, and she had only mild anemia.

## Discussion

The differential diagnosis of anorexia nervosa includes other eating and psychiatric disorders with similar symptoms, such as avoidant/restrictive food intake disorder, unipolar major depression, obsessive-compulsive disorder, body dysmorphic disorder, attention deficit hyperactivity disorder, and social phobia. Patients with these illnesses are often concerned about weight loss, do not restrict their caloric intake and their body image is not distorted. However, in patients with anorexia nervosa, depression and anxiety are common psychiatric comorbidities [3]. There are several reports that neuropsychiatric symptoms, including depression and anxiety, may be present in patients with celiac disease. Several mechanisms have been suggested to explain this association. Metabolic disorders due to malabsorption and emotional stress related to celiac disease diagnosis are risk factors. Another potential pathogenic mechanism is the low levels of tryptophan in patients with untreated celiac disease. Tryptophan is a precursor of serotonin synthesis and low tryptophan concentrations in the brain lead to depression [10].

Patients with suggestive gastrointestinal symptoms should be tested for celiac disease. The group of patients with high celiac disease probability includes individuals with highly suggestive symptoms such as chronic diarrhea or steatorrhea and weight loss, and individuals with moderate to high risk of celiac disease and consistent gastrointestinal or extraintestinal symptoms such as iron deficiency anemia and persistent elevation in serum aminotransferases. Risk factors associated with moderate to high risk for celiac disease include first- and second-degree relatives with celiac disease, type 1 diabetes, autoimmune thyroiditis, and Down and Turner syndrome [11].

Observational studies suggest that females with anorexia nervosa have an elevated risk of a bidirectional association between anorexia nervosa and autoimmune diseases, particularly Addison's disease, celiac disease, myxoedema, ulcerative colitis, and pernicious anemia [7]. In addition, a Swedish Register-based cohort showed that patients with celiac disease are at increased risk of anorexia nervosa before or after diagnosis of celiac disease [8]. Potential explanations for this association include a diverse genetic link between celiac disease and anorexia nervosa, which involves *HLA* haplotypes and other possible immunological mechanisms, including brain-reactive autoantibodies and increased pro-inflammatory cytokine production. The overproduction of pro-inflammatory cytokines in the brain can lead to the activation of the HPA (hypothalamic-pituitary-adrenal) axis, whose role in the pathogenesis of anorexia nervosa has been established. Brain-reactive antibodies are present in autoimmune diseases and crossreact with neurotransmitters and hormonal regulators of appetite, and this may lead to disturbed appetite and decreased intake of food. [9].

Similar gastrointestinal and extraintestinal symptoms characterize both celiac disease and anorexia nervosa, and it is sometimes difficult to confirm the diagnosis. Our patient also had elevated aminotransferases. Mild to moderate elevation in liver chemistry tests is a common laboratory finding and has been described in approximately 47 percent of patients with biopsy-proven celiac disease [12]. In patients with untreated celiac disease, the intestinal barrier becomes more permeable and allows toxins to enter the systemic circulation and reach the liver through the portal circulation, resulting in liver injury. Aminotransferase levels normalized in 95% of the patients after one year of a strict gluten-free diet [13]. A rise in aminotransferases is not an uncommon laboratory finding in patients with anorexia nervosa. The pathophysiologic mechanism of liver dysfunction is starvation-induced autophagy, which leads to hepatocyte injury and death [14]. The alanine transaminase: aspartate transaminase (ALT: AST) ratio in celiac disease usually is less than 1 [13]. In contrast, the ALT: AST ratio is greater than 1 in anorexia nervosa, showing a higher level of ALT activity [14], and consequently may be a useful diagnostic tool to differentiate celiac disease from anorexia nervosa.

## Conclusions

Our case highlights the importance of further investigation in patients with eating disorders who complain of persistent abdominal symptoms. The additional work-up depends on the medical history. A history of autoimmune disease should raise further suspicion of celiac disease. A physician should know that similar gastrointestinal and extraintestinal symptoms characterize both celiac disease and anorexia nervosa. Several simple laboratory tests, such as the ALT: AST ratio, may be helpful differential diagnostic tools.

## References

[1] Court JP, Kaplan AS (2016). The disjointed historical trajectory of anorexia nervosa before 1970. Curr Psychiatry Rep.

[2] Call C, Walsh BT, Attia E (2013). From DSM-IV to DSM-5: changes to eating disorder diagnoses. Curr Opin Psychiatry.

[3] Keel PK, McCormick L (2010). Diagnosis, assessment, and treatment planning for anorexia nervosa. The treatment of eating disorders: a clinical handbook.

[4] Marzola E, Nasser JA, Hashim SA, Shih PA, Kaye WH (2013). Nutritional rehabilitation in anorexia nervosa: review of the literature and implications for treatment. BMC Psychiatry.

[5] Lindfors K, Ciacci C, Kurppa K (2019). Coeliac disease. Nat Rev Dis Primers.

[6] Cichewicz AB, Mearns ES, Taylor A (2019). Diagnosis and treatment patterns in celiac disease. Dig Dis Sci.

[7] Raevuori A, Haukka J, Vaarala O (2014). The increased risk for autoimmune diseases in patients with eating disorders. PLoS One.

[8] Mårild K, Størdal K, Bulik CM, Rewers M, Ekbom A, Liu E, Ludvigsson JF (2017). Celiac disease and anorexia nervosa: a nationwide study. Pediatrics.

[9] Wotton CJ, James A, Goldacre MJ (2016). Coexistence of eating disorders and autoimmune diseases: record linkage cohort study, UK. Int J Eat Disord.

[10] Ludvigsson JF, Reutfors J, Osby U, Ekbom A, Montgomery SM (2007). Coeliac disease and risk of mood disorders--a general population-based cohort study. J Affect Disord.

[11] Ludvigsson JF, Card TR, Kaukinen K, Bai J, Zingone F, Sanders DS, Murray JA (2015). Screening for celiac disease in the general population and in high-risk groups. United European Gastroenterol J.

[12] Jacobsen MB, Fausa O, Elgjo K, Schrumpf E (1990). Hepatic lesions in adult coeliac disease. Scand J Gastroenterol.

[13] Villavicencio Kim J, Wu GY (2021). Celiac disease and elevated liver enzymes: a review. J Clin Transl Hepatol.

[14] Rosen E, Bakshi N, Watters A, Rosen HR, Mehler PS (2017). Hepatic complications of anorexia nervosa. Dig Dis Sci.

